# Combining PET Images and Neuropsychological Test Data for Automatic Diagnosis of Alzheimer's Disease

**DOI:** 10.1371/journal.pone.0088687

**Published:** 2014-02-13

**Authors:** Fermín Segovia, Christine Bastin, Eric Salmon, Juan Manuel Górriz, Javier Ramírez, Christophe Phillips

**Affiliations:** 1 Cyclotron Research Centre, University of Liège, Liège, Belgium; 2 Department of Signal Theory, Networking and Communications, University of Granada, Granada, Spain; 3 Department of Electrical Engineering and Computer Science, University of Liège, Liège, Belgium; The University of Chicago, United States of America

## Abstract

In recent years, several approaches to develop computer aided diagnosis (CAD) systems for dementia have been proposed. Some of these systems analyze neurological brain images by means of machine learning algorithms in order to find the patterns that characterize the disorder, and a few combine several imaging modalities to improve the diagnostic accuracy. However, they usually do not use neuropsychological testing data in that analysis. The purpose of this work is to measure the advantages of using not only neuroimages as data source in CAD systems for dementia but also neuropsychological scores. To this aim, we compared the accuracy rates achieved by systems that use neuropsychological scores beside the imaging data in the classification step and systems that use only one of these data sources. In order to address the small sample size problem and facilitate the data combination, a dimensionality reduction step (implemented using three different algorithms) was also applied on the imaging data. After each image is summarized in a reduced set of image features, the data sources were combined and classified using three different data combination approaches and a Support Vector Machine classifier. That way, by testing different dimensionality reduction methods and several data combination approaches, we aim not only highlighting the advantages of using neuropsychological scores in the classification, but also implementing the most accurate computer system for early dementia detention. The accuracy of the CAD systems were estimated using a database with records from 46 subjects, diagnosed with MCI or AD. A peak accuracy rate of 89% was obtained. In all cases the accuracy achieved using both, neuropsychological scores and imaging data, was substantially higher than the one obtained using only the imaging data.

## Introduction

Dementia is one of the most common neurodegenerative disorders in elderly and it is expected that its prevalence increases in the near future, mainly due to the aging population in developed nations [1]. An early and accurate diagnosis will allow patients to benefit from new treatments or strategies that may delay the progress of the disease [2]–[7]. In recent years, many computer-aided diagnosis (CAD) systems for neurodegenerative disorders have been presented [5], [8]–[10]. Based on the assumption that pathological manifestations of these disorders appear some years before subjects become symptomatic [11], [12], they try to diagnose them even before the classical diagnosis procedure based on neuropsychological tests does.

Several approaches have been used to develop a CAD system for dementia. The most familiar approach to the neuroimaging community is univariate statistical testing which analyzes separately each voxel of the brain images, for example performed with the Statistical Parametric Mapping (SPM) [13] package. Such univariate processing can somehow also be used for diagnosis by comparing the subject under study and the control group [4], [6], [14]. On the other hand, multivariate approaches analyze all the voxels together, taking into account the relations between voxels to output a prediction [10], [15], [16]. The growth of the multivariate systems is mostly due to the recent advances on machine learning [17] which provide more reliable statistical classifiers, with a higher ability to address the small sample size problem [18]. This problem can also be addressed by means of a feature extraction technique that reduces the huge amount of data contained in a brain image into a relatively small unidimensional vector. In this case, the structure of the CAD systems based on neuroimaging and machine learning is as follows: After the preprocessing of the images (which involves the spatial registration and the intensity normalization), an algorithm is applied to select and summarize the relevant information. This information is rearranged in a vector and used as feature for the classification step. Finally, a classifier is used to separate pathological and control subjects. In terms of neuroimaging modalities, researches have used both structural [2], [19] and functional data [9], including nuclear imaging modalities such as PET [5], [20] and SPECT [3], [7].

In this work, we study the benefits of taking into account the information derived from neuropsychological tests in the development of computer systems to aid the diagnosis of dementia. Recently, some studies that combine data from different image modalities, even that include biological measures such as cerebrospinal fluid (CSF) assays [21] have been presented, but the use of neuropsychological scores along the imaging data have not yet been fully explored. We hypothesized that using such information in the development of CAD systems for dementia will improve their accuracy since neuropsychological testing is of great importance for identifying the cognitive profiles characteristic of a diagnosis [22] and, in fact, it has been classically used to diagnose the dementia. In addition, neuropsychological tests are relatively inexpensive and totally innocuous for the patients, compared to nuclear medicine imaging. In order to validate this hypothesis, we evaluated the accuracy of several CAD systems for AD. Specifically, the developed systems attempt to distinguish patients with stable Mild Cognitive Impairment (MCI) from those whose disease evolves to AD in the next few years, who therefore may be considered as “early AD”. Several approaches were used to combine the information from neuropsychological tests and functional brain images. In addition, three dimensionality reduction methods were applied to the images before the combination, pursuing two goals. On the one hand, the reduction allows to overcome the small sample size problem and, on the other hand, it allows to address the large difference between the dimensionality of one image and the number of neuropsychological scores for one subject. By means of a leave-one-out cross-validation scheme, the accuracy rates obtained by these systems were estimated and compared with the ones obtained by similar systems that only use the imaging data or the neuropsychological scores in the classification.

## Materials and Methods

### Ethics Statement

Each patient (or a close relative) gave written informed consent to participate in the study and the protocol was accepted by the University Ethics Committee in Liege. All the data were anonymized by the clinicians who acquired them before being considered in this work. Nowadays, the data are hosted in the Cyclotron Research Centre (Belgium) but they will be entered to the European Alzheimer Disease Consortium database (please visit www.eadc.info for further information) once published. That way the data will be available for the scientific community.

### Database

A database collected during a recent longitudinal study was used to evaluate our proposed approach. It includes data from 46 subjects who were originally diagnosed with MCI (see the demographic details in Table 1): one Positron Emission Tomography (PET) image and five neuropsychological scores were acquired per subject. In addition the Mini Mental State Examination (MMSE) score [23] and the age of the patients were considered. The acquisition of the PET images were performed 30 minutes after injection of the ^18^F-FDG radiopharmaceutical, by means of a Siemens CTI 951 R 16/31 gamma camera. Three neuropsychological scores were derived from a verbal cued recall memory task, reflecting respectively the efficiency of memory encoding (immediate recall), long-term episodic memory (cued recall) and monitoring capacities (intrusions). This task, that provides support at both encoding and retrieval, previously proved to efficiently discriminate between healthy older adults and AD patients, as well as between stable MCI and converters [24], [25]. The other two neuropsychological scores were phonemic (letter P) and semantic (animals) verbal fluency measures, as an index of executive functioning. These measures were included as impaired executive functioning and semantic memory are also sensitive markers of decline in MCI [26], [27].

**Table 1:** Database details.

	MCI stable	MCIbecome AD	Between-group differences
Age	65.55±7.76	72.42±5.91	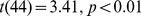
Education (years)	11.95±3.44	11.38±4.40	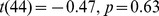
MMSE	27.10±1.62	25.26±2.76	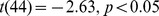
Gender (M/F)	10/10	11/15	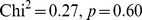

Demographic details of the subjects who participated in this study. Average and standard deviation are given respectively for age, education and MMSE.

**Figure 1:**
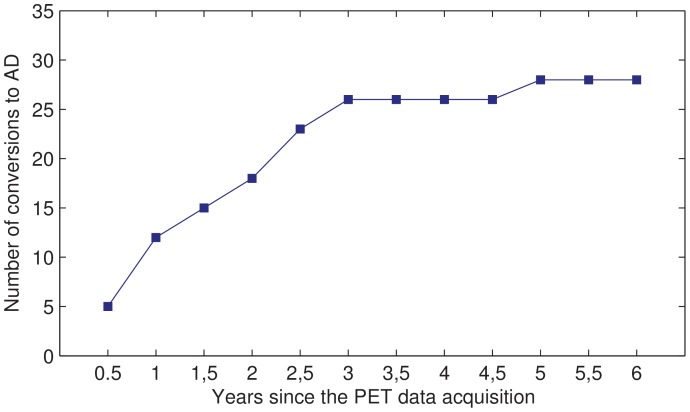
Evolution of the patients' diagnosis. Number of subjects whose diagnosis changed to AD during the 6 years after the beginning of the study.

The subjects were monitored during the following years and neuropsychological tests were repeated periodically. Based on these periodical tests, the diagnosis of some patients changed to AD. In order to label the initial data (PET images and neuropsychological scores from the first diagnosis) as pertaining to stable MCI or (early) converter, and taking into account the fact that even patients who were stable several years after inclusion may develop AD at some unknown point in the future, a time limit to consider the conversions should be fixed. Figure 1 shows the evolution of the diagnosis of the studied subjects during the 6 years after the database creation. As can be noticed, there were a lot of conversions during the first 3 years but later on, the number of MCI subjects decreased in a much slower way. The initial data were therefore labeled using the diagnosis after 3 years: 26 subjects were labeled as “MCI become AD” or “AD” for short, whereas the remaining 20 subjects were labeled as “MCI stable” or simply “MCI”. The study therefore focuses on early converters, consistently with the interest of detecting relatively fast decline in clinical practice.

After the acquisition and a proper reconstruction, all the PET images were spatially normalized using the template matching approach implemented in SPM5 [13], [28], [29]. In order to ensure an accurate normalization of our images from old adults, the normalization procedure was run twice. Firstly, using the template provided by SPM5 (built with images from young healthy adults) and, secondly, using an *ad hoc* template computed as the average of all our images (after the first spatial normalization). The intensity normalization was performed by scaling the intensities of each image with respect to the intensity values obtained in the cerebellum. According to a recent study [30], this method is superior to global normalization in identifying dementia patients in comparison to control subjects. The cerebellar region was delimited by means of the automatic anatomical labeling atlas (AAL) [31], in a way similar to the procedure performed in [32].

### Image Dimensionality Reduction

An important issue that should be addressed in the computerized analysis of neuroimages is the so called small sample size problem [18]: The high dimensionality of that kind of images related to the (relatively low) number of images included in the studies can lead to overfitting and poor generalization performances. This problem can be addressed by means of dimensionality reduction techniques that summarize the information contained in the images [5], [33], [34]. In this work, three dimensionality reduction algorithms based on several classical techniques were considered:

#### Dimensionality reduction based on Principal Component Analysis (PCA)

PCA [35] is a mathematical procedure that rotates the axes of data space along the lines of maximum variance. The axis of greatest variance are called principal components. The dimensionality reduction of 3D images based on PCA may be performed as follows [7]: Let 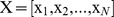 be a set of *N* functional brain images in vector form. After normalizing the images to unity norm and subtracting the mean, a new set 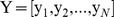 is obtained. The covariance matrix of the normalized vectors set is defined as:
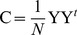
(1)


Then, the eigenvector 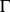 and eigenvalue 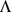 matrices are computed as 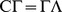. Since the image size is greater than the number of images, diagonalizing 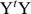 instead of 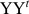 reduces the computational burden and the eigenvectors/eigenvalues decomposition is reformulated as [36]:
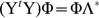
(2)




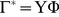
(3)where 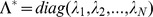 and 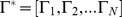 are the first *N* eigenvalues and eigenvectors respectively. Finally, the images are modeled by projecting them over those eigenvectors (a.k.a. principal components).

#### Dimensionality reduction based on Partial Least Squares (PLS)

PLS is a statistical method for modeling relations between sets of observed variables by means of latent variables [37]. The underlying assumption is that the observed data is generated by a system or process which is driven by a small number of latent (not directly observed or measured) variables. In that sense, it is similar to PCA (in fact, both are based on the singular value decomposition) however PLS performs the decomposition so that covariance between the data and a set of properties of the data is maximum. Mathematically, PLS is a linear algorithm for modeling the relation between two data sets 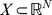 and 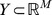. After observing *n* data samples from each block of variables, PLS decomposes the 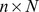 matrix of zero-mean variables, **X**, and the 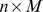 matrix of zero-mean variables, **Y**, into the form:
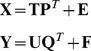
(4)where the **T** and **U** are 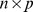 matrices of the *p* extracted score vectors (also known as components or latent vectors), the 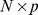 matrix **P** and the 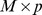 matrix **Q** are the matrices of loadings and the 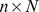 matrix **E** and the 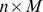 matrix **F** are the matrices of residuals (or error matrices).

PLS may be used for dimensionality reduction of PET images by performing the decomposition of the intensity values (matrix 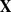) and the image labels (matrix 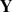). The 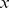-scores in **T** are linear combinations of the 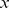-variables and can be considered as a good summary of 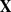. In addition, performing the composition that way maximizes the covariance between the images and their labels, thus 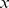-scores contains the relevant information for a further classification step [5].

#### Dimensionality reduction based on Independent Component Analysis (ICA)

ICA is a computational method to express a set of random variables as linear combinations of statistically independent component variables. Its main applications are blind source separation and feature extraction. In its linear form, the problem consists on finding the sources 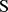 which, when mixing using a weight matrix 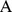, provide the vector 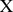 of observed variables:
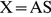
(5)where the sources 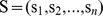 are assumed to be statistically independent. In order to estimate both the mixing matrix 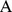 and the sources 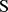, ICA adaptively calculates the matrix 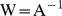 which either maximizes the nongaussianity or minimizes the mutual information [34]. This technique has been successfully applied to dimension reduction problems by projecting the data into its independent components, performing that way the reduction [34].

PCA-, PLS- and ICA-based methodologies allow the reduction of a neuroimage to a vector of scores which size is related to the total number of images used in the study. A further reduction of the image dimensionality may be performed by selecting only the most important scores or components. The importance of the components may be estimated through several methods. In this work, the importance of the PCA components was estimated by their contribution to the total variance of the image set. Specifically, we selected as few components as possible to gather 75% of the total variance. This threshold was estimated through cross-validation to get the highest accuracy in the subsequent classification procedure. Similarly, we used cross-validation to select which scores/components would be taken into account with the PLS and ICA approaches. However, in these methods, the variance does not plays the same role as in PCA, thus we used the Fisher Discriminant Ratio (FDR) instead. Specifically, we selected as few components as possible so that the sum of the FDR of the selected components is 85% of the total FDR (the sum of the FDR value of all the components). As in the PCA case, this percentage was selected through cross-validation.

FDR [38] is a separability criterion derived from Fisher Discriminant Analysis (FDA) and widely used in pattern recognition problems [7], [39], [40]. Its main idea can be briefly described as follows. Suppose that there are two kinds of sample points in a 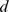-dimension data space. FDR is a measure of the separability between the points of two classes when you project the data over a given direction in the original space. For feature selection purposes, the following formula is first applied to each feature and then the features with highest FDR value are selected:
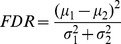
(6)where 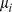 and 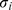 denote, respectively, the mean and the variance for the 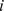-th class samples.

### Combining PET Data and Neuropsychological Scores

**Figure 2:**
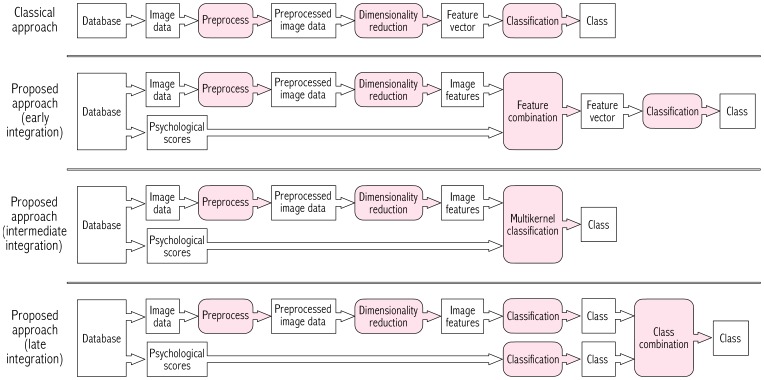
Data flow of CAD systems for neurodegenerative disorders. Comparison between the classical approach used in most part of CAD systems for AD and the proposed approach, which consists of taking into account the neuropsychological test data along the brain images. Last three rows show the differences between the ways of integrating in the system the data from the neuroimages and from the neuropsychological tests.

Brain PET images and neuropsychological tests provide information of different nature (values are in different range and should be interpreted in a different manner) and the combination of both sources should accounts for this. According to the literature, the combination of heterogeneous data sources in a classification procedure may be performed at three levels [41], [42]: before, during or after the classification step. These three theoretical approaches, illustrated in Figure 2, have been implemented in this work as follows:

#### Early integration

The information from both sources is combined before the classification step into a single feature vector per subject. This vector contains the neuropsychological scores and the image features, i.e. the result of applying one of the dimensionality reduction methods described above to the brain image. Specifically, the feature vector is built by concatenating the neuropsychological scores (including MMSE and age where appropriate) and the image features:
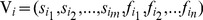
(7)where 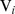, 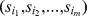 and 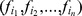 are, respectively, the feature vector, the neuropsychological scores and image features for subject 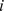.

#### Intermediate integration

In this approach, a.k.a. multikernel classification [42], the combination is performed inside the classifier by using two kernel matrices, one per data source. A key question in this approach is the way in which the kernel matrices are combined. Linear [43], [44], non-linear [45], [46] and data-dependent [47], [48] approaches have been proposed. Here, we propose to apply a linear weighted function, which works fine in experiments with small databases like the one used in this work. The combination function is:
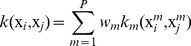
(8)where 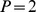 is the number of kernels; 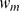 stand for the weight for kernel 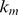; 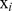, 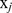 are two feature vectors and 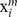, 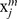 are subset of 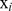, 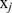 with only the features used for kernel 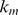.

#### Late integration

An individual classifier is used for each data source, and the final output prediction is estimated by combining the outputs of all the classifiers. This combination is performed by considering the confidence of each estimation. Since we used Support Vector Machine (SVM) [49] classifiers, the confidence of the estimations were computed by means of the distance to the maximal margin hyperplane. Specifically, two classes, 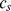 and 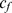, were estimated for each subject using respectively the neuropsychological scores 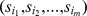 and the image features 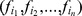. Along the class labels, the distances to the separation hyperplanes defined by the classifiers, 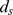 and 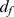, were computed. Finally, the class 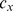 such that 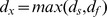, 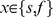 was taken into account; the other one was discarded. In the (very unlikely) case of 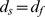, the class corresponding to the classification with higher accuracy in individual experiments (using only one data source) was selected.

## Results

In order to not only measure the improvements of using neuropsychological testing data along the images, but also find the most accurate CAD system for early AD, all possible combinations of dimensionality reduction methods and data sources integrations were evaluated. For the classification step, a SVM classifier was used as done for similar problems [5], [7], [9]. The accuracy rates, gathered in the table 2, were estimated through a leave-one-out procedure. In all cases, the parameters needed were computed by maximizing the accuracy in a previous cross-validation loop. For example, for the cost parameter of the SVM classifier, 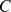, values of 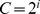 with 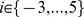 were used. For the kernel, linear and non-linear functions were tested. Except for the multikernel approaches, the classifiers using a linear kernel always outperformed those with a non-linear kernel. In the late integration approach, we used the classification parameters that had achieved the best results in the individual experiments (only images and only neuropsychological scores) for each of the two classifiers.

**Table 2:** Classification rates.

Approach	Dim. red.method	Accuracy	Sensitivity	Specificity	Positive	Negative
					Likelihood	Likelihood
	PCA	73.91%	76.92%	70.00%	2.56	0.33
Only images	PLS	78.26%	84.62%	70.00%	2.82	0.22
	ICA	69.57%	76.92%	60.00%	1.92	0.38
Only psych. scores	–	73.91%	73.08%	75.00%	2.92	0.36
Psych. sc.+MMSE+Age	–	84.78%	84.62%	85.00%	5.64	0.18
Images and	PCA	80.43%	84.62%	75.00%	3.38	0.21
psych. scores	PLS	84.78%	88.46%	80.00%	4.42	0.14
(Early integration)	ICA	76.09%	80.77%	70.00%	2.69	0.27
Images and psych.	PCA	80.43%	84.62%	75.00%	3.38	0.21
scores+MMSE+Age	PLS	84.78%	88.46%	80.00%	4.42	0.14
(Early integration)	ICA	73.91%	76.92%	70.00%	2.56	0.33
Images and	PCA	82.61%	92.31%	70.00%	3.08	0.11
psych. scores	PLS	84.78%	88.46%	80.00%	4.42	0.14
(Interm. integration)	ICA	82.61%	84.62%	80.00%	4.23	0.19
Images and psych.	PCA	89.13%	92.31%	85.00%	6.15	0.09
scores+MMSE+Age	PLS	89.13%	92.31%	85.00%	6.15	0.09
(Interm. integration)	ICA	89.13%	92.31%	85.00%	6.15	0.09
Images and	PCA	86.96%	92.31%	80.00%	4.62	0.10
psych. scores	PLS	84.78%	88.46%	80.00%	4.42	0.14
(Late integration)	ICA	80.43%	80.77%	80.00%	4.04	0.24
Images and psych.	PCA	89.13%	92.31%	85.00%	6.15	0.09
scores+MMSE+Age	PLS	89.13%	92.31%	85.00%	6.15	0.09
(Late integration)	ICA	89.13%	92.31%	85.00%	6.15	0.09

Accuracy, sensitivity, specificity and positive and negative likelihoods for the systems implemented. These rates were estimated by means of a leave-one-out cross-validation scheme and using the database described above.

All the experiments that involve the neuropsychological scores were run twice: using only the five neuropsychological scores described above and including the MMSE score and the age as two additional neuropsychological scores. That way, it is possible to measure the influence of those five neuropsychological scores in the classification.

**Figure 3:**
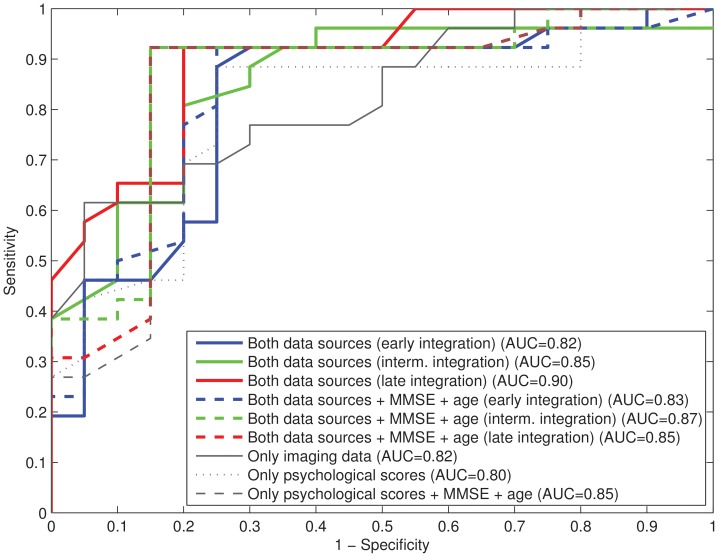
Comparison of the trade off between sensitivity and specificity. ROC curves for the three cases analyzed: using only images, using only neuropsychological scores and using both images and neuropsychological scores (including three approaches: early, intermediate and late integration). The area under the curve (AUC) is shown in the legend.

In order to highlight the difference between the solutions using or not neuropsychological scores, MMSE score and age, a further comparison was performed by means of the Receiver Operating Characteristic (ROC) curves for the three approaches: using only the neuropsychological scores, using only the imaging data and using both the neuropsychological scores and the imaging data (see figure 3). A ROC curve is a plot of the trade off achieved between sensitivity and specificity for a classification procedure. The optimal solution is located in the upper left corner and corresponds to a sensitivity and specificity of 100%. Therefore the closer the ROC curve is to the upper left corner, the higher the overall accuracy of the procedure [50]. A value to measure this accuracy is provided by the area under the curve (AUC).

**Figure 4:**
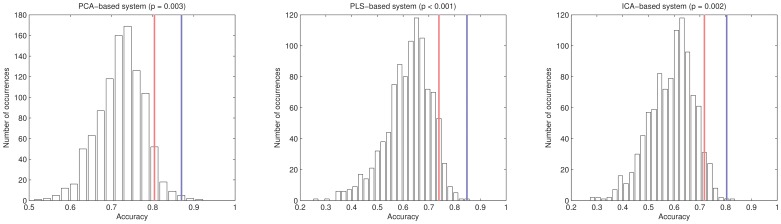
Histograms of the non-parametric test. Histogram of the accuracy rates achieved by using randomly generated neuropsychological scores (1000 repetitions) and the late integration approach. Red lines are the accuracies associated with a 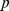-value of 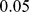. Blue lines are the accuracies for the late integration approach reported in table 2.

Finally, a non-parametric test [51] was performed to assess the statistical difference between the accuracy rates obtained by the proposed and the previous approaches, i.e. by using neuropsychological scores beside the imaging data or only the imaging data. 1000 sets of random neuropsychological scores (same range as the original ones) were generated, then classifier was trained with these random scores (and the image features) and the accuracy estimated. The histograms for all the PCA-, PLS- and ICA-based systems are shown in Figure 4. A 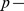
value was then calculated as the number of cases where the accuracy obtained with the random scores was larger than that obtained with the true scores, divided by 1000, i.e. the probability of obtaining a better accuracy with a random score. As the result, 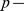
values of 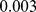, 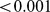 and 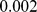 were obtained for the PCA-, PLS- and ICA- based CAD systems respectively.

## Discussion

The aims of this work were, in the one hand, to measure the advantages of using neuropsychological testing data in the neuroimaging-based CAD systems (which usually use only imaging data) for neurodegenerative disorders and, on the other hand, to develop the more accurate CAD system for early AD to date. In light of the results shown in table 2, we can say that taking into account the information from neuropsychological tests improves the accuracy of the analyzed CAD systems. That improvement is achieved by using different ways of combining the data and does not depend on the processing applied to the neuroimages, i.e. the dimensionality reduction algorithm employed. For example, the PCA-based systems provide an accuracy of 73.9% when only images are used, and an accuracy close to 90% when neuropsychological scores, MMSE and age are taken into account. Even when MMSE and age are not used, the accuracy of the systems is about 10% higher using both sources than using only imaging data. This fact also corroborates the validity for the diagnosis of the five neuropsychological scores described in the material and methods section. It is worth noting that the improved accuracy is due to higher value of both sensitivity and specificity rates, achieving a good balance between these measures when the data from both sources is included.

Regarding the comparison of using only neuropsychological scores or using neuropsychological scores and imaging data, two cases are considered. On the one hand, if the MMSE score and the age are used in the classification, there is no large difference in terms of accuracy between both approaches. In fact, the accuracy rate for the early integration methodology is smaller or equal than the one for using only neuropsychological scores. For intermediate and late integration approaches, the inclusion of the imaging data provides an increase of accuracy about 5%. The differences between the combination methods are due to the nature of them. Whereas in intermediate and late integration, both data sources have a priori similar weights in the final decision, in early integration the weight of imaging data is usually higher since the number of features related to them is larger than the number of neuropsychological scores (see equation 7). The high accuracy rates achieved in general when MMSE and age are added in the classification are due to the significant differences between our groups that exist in these two variables (as shown in table 1). In some sense, this is a limitation of the data used and unfortunately cannot be corrected in a multivariate analysis [52]. On the other hand, if the MMSE score and the age are not included in the classification the combination of both, neuropsychological scores and imaging data, provide an increase about 10% in the accuracy of the systems.

The second objective is not easy to verify since the comparison between the accuracy rates highly depend on the database used to estimate them, and small differences may not be statistically significative (in our case, all the AD subjects are borderline subjects and, in fact, they had been diagnosed with MCI a short time before). Nevertheless a rough comparison may be drawn with some previous works. In [53] the authors classify MCI converter versus MCI non-converter using magnetic resonance imaging (MRI) and cerebrospinal fluid biomarkers. The accuracy reported is about 60%, distinctly lower than the one achieved in this work. In [54] a multimodal approach that uses MRI, diffusion tensor and PET imaging to separate MCI and AD subjects is presented. They obtained a peak accuracy of 73.5%, also far from the peak accuracy rates achieved in this work. Finally, in [55] an accuracy rate about 75–80% (with a maximum of 81.5%) is reported when they classify MCI converter versus MCI non-converter from the ADNI database. These results are near to the ones obtained here, however the inclusion of the neuropsychological testing data allowed to achieve average rates over 80% and higher peak values.

Regarding the combination approaches, we should evaluate them not only in terms of accuracy but also in terms of efficiency and simplicity. In terms of accuracy, the intermediate and late integration methodologies yield similar rates, with peak values of 89%, whereas the peak value for the early integration approach is close to 85%. Anyway, where the MMSE score and the age are not considered the differences between three approaches are smaller and the early integration approach has the advantage of being the simplest one.

The ROC analysis (Figure 3) provides other way of measuring the differences of using both data sources together. This figure confirms the higher accuracy of the methods that consider the neuropsychological testing data and shows that they provide an adequate trade-off between sensitivity and specificity. The non-parametric test allows to compute significance measures (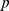-values) for the classification procedure. Specifically, it estimates if the probability of the improvement achieved by introducing the neuropsychological scores is due to chance. The obtained values (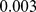, 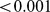 and 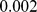 for systems based on PCA, PLS and ICA respectively) discard this possibility and confirms the interest of combining imaging and neuropsychological data for differentiating our patients' groups, instead of using only imaging data.
